# Patient and public involvement in an international rheumatology translational research project: an evaluation

**DOI:** 10.1186/s41927-022-00311-w

**Published:** 2022-10-22

**Authors:** Savia de Souza, Eva C. Johansson, Susanne Karlfeldt, Karim Raza, Ruth Williams

**Affiliations:** 1 Centre for Rheumatic Diseases, King’s College London, London, SE5 9RJ UK; 2 Swedish Rheumatism Association (Reumatikerförbundet), Box 90337, 120 25 Stockholm, Sweden; 3 Rheumatology Unit, Karolinska Institutet and Academic Specialist Center, Stockholm Health Services, 171 77 Stockholm, Sweden; 4 Institute of Inflammation and Ageing, University of Birmingham, Birmingham, B15 2WB UK

**Keywords:** Patient and public involvement, Patient engagement, Rheumatoid arthritis, Evaluation, Surveys, Clinical trials, Translational research

## Abstract

**Background:**

Rheuma Tolerance for Cure (RTCure) is a five-year international collaboration between academia, industry and patients/members of the public. It focuses on developing approaches to predict the onset of rheumatoid arthritis (RA) and designing clinical trials to reduce the risk of disease development through immune-tolerising and other treatments. We conducted a mid-term evaluation of patient and public involvement (PPI) within the project.

**Methods:**

Two surveys on PPI were co-designed by the PPI Coordinator, Patient/Public Research Partners (PRPs) and a researcher. Both anonymous, electronic surveys were distributed to 61 researchers and 9 PRPs. Quantitative survey data were analysed using descriptive statistics and free text responses underwent inductive thematic analysis.

**Results:**

Researcher and Patient response rates were 33% and 78%, respectively. Quantitative Researcher Survey data highlighted that (i) responding researchers represented all seven Work Packages (WPs), (ii) 40% thought PRPs had made a large or extremely large contribution to their own WPs, (iii) 55% thought PPI has had a moderate or large impact on RTCure, (iv) 75% worked with PRPs in RTCure, and (v) 60% said PRPs had affected their research thinking. Quantitative PRP Survey data highlighted that (i) PRPs were most involved in four WPs, (ii) 43% thought they had made a minor contribution to their main WP, (iii) 57% thought PPI has had a small impact on RTCure, and (iv) 57% thought they received too little feedback on the outcome of their contribution to different tasks. Four main themes were identified in both surveys: ‘PRP contributions’, ‘Experiences of PPI’, ‘Impact of PPI on RTCure’, and ‘How PPI can be improved’. Two additional themes from the Researcher Survey were ‘Impact of PPI on researchers’ and ‘Influence on Future Projects’, and from the PRP Survey were ‘Impact of PPI on PRPs’ and ‘Engagement with PRPs’.

**Conclusion:**

PPI seemed to have a significant impact on RTCure, however, PRPs were less aware. A focus on improving communication between PRPs and researchers (facilitated by the PPI Coordinator), and providing PPI training for researchers is likely to improve involvement. Complex legal agreements for PRPs should be avoided and careful attention paid to appropriate PRP compensation.

**Supplementary Information:**

The online version contains supplementary material available at 10.1186/s41927-022-00311-w.

## Background

Patient and public involvement (PPI) in research has been defined as “research being carried out ‘with’ or ‘by’ members of the public rather than ‘to’, ‘about’ or ‘for’ them”; it is different to ‘participation’ where people partake in a research study, and ‘engagement’ where research information and knowledge is provided and disseminated [1]. Over the last few decades, PPI has been increasingly utilized in healthcare settings [2–4], with examples in rheumatology including its use in clinical trials [5–10] and to improve services [3, 11–16]. PPI has been shown to improve clinical trial design and increase patient enrolment [17, 18]. However, a recent analysis of journal articles from 2016 to 2020 found that patients were only involved in 1.8% of rheumatology clinical trials reported during that period; none of these were industry-initiated [19].

It is recommended by the European League Against Rheumatism (EULAR) that at least two patient representatives are included in scientific projects and are involved in all project phases [20]. It is especially important to have PPI in rheumatology as rheumatic conditions are often lifelong and life-changing [21]. The Innovative Medicines Initiative (IMI), a European Union (EU) public–private partnership funding health research and innovation [22], encourages patient involvement in all their activities [23] as do the European Federation of Pharmaceutical Industries and Associations (EFPIA) [24].

Rheuma Tolerance for Cure (RTCure) is a large-scale, IMI-funded international collaboration between academia, industry, patients and members of the public, with a focus on early detection and prevention of rheumatoid arthritis (RA). This translational research project aims to develop immune-tolerising and other treatments, and develop and conduct clinical trials to reduce the risk of developing RA [25]. RTCure was initially conceived in December 2015, with the final submission to the funder in July 2017. It commenced in September 2017 with a kick-off meeting and was due to complete in August 2022, but is likely to be extended until August 2023 due to the impact of coronavirus disease 2019 (COVID-19).

The RTCure consortium includes researchers from 12 academic institutions in 9 countries (Australia, Austria, Belgium, Germany, Hungary, The Netherlands, Sweden, Switzerland, United Kingdom), 6 pharmaceutical companies and 2 small-medium enterprises; and is coordinated by the Karolinska Institute in Sweden. The project is divided into seven Work Packages (WPs): WP1—management, coordination, dissemination and sustainability; WP2—cohorts; WP3—mechanisms of immune tolerance; WP4—technologies for monitoring the RA-associated immune state; WP5—bioinformatics and data; WP6—clinical studies; and WP7—ethical requirements [25]. Further details on the WPs can be found at the end of Additional files 1 and 2.

Nine Patient/Public Research Partners (PRPs) from five countries (Germany, The Netherlands, Romania, Sweden, United Kingdom) are involved in RTCure (6 females: 3 males; 7 Caucasians: 2 minority ethnics; 8 with established RA: 1 ‘at-risk’ of RA). The PRPs work across all the WPs to varying degrees. Patient Research Partners are defined as ‘persons with a relevant disease who operate as active research team members on an equal basis with professional researchers, adding the benefit of their experiential knowledge to any phase of the project.’ [20]. We decided to use the term Patient/Public Research Partner as one of our PRPs is ‘at-risk’ of developing RA and therefore not defined as a patient.

In order to understand researcher and PRP views of PPI within RTCure, and to assess whether additional changes needed to be made to improve it, we conducted a mid-term evaluation of PPI. This was originally due to take place in June 2020 but was delayed until January 2021 due to the COVID-19 pandemic. Whilst PPI impact is often measured quantitatively [26], qualitative work has provided examples of situations in which even when PRPs are involved from the agenda setting stage of research they have limited impact on the management and implementation of the research thereafter [27]. So, it is also important that the experiential and contextual impact of PPI on researchers and PRPs is captured [28]. Therefore, we chose to include open-ended questions in our surveys.

We hope that our findings will influence future researcher-PRP collaborations beyond RTCure, particularly in relation to preclinical/experimental medicine research and research with significant involvement of the pharmaceutical industry, where knowledge about optimal approaches to PPI is limited.

## Methods

### PPI in RTCure

In late 2017/early 2018 PRPs with or ‘at-risk’ of developing RA were recruited via national patient organisations, the EULAR Patient Research Partner Network and clinical researchers within the consortium. There was no formal selection process, with all interested PRPs being included in the project. Six PRPs had previous involvement experience whilst three did not; although they were signposted towards EULAR PPI training resources, no project-specific PPI training was provided.

RTCure has a PPI Coordinator [SK] (appointed by the consortium for the project as a 0.25 whole time equivalent post). Her role is to help develop the PRP group, aid communication between PRPs and researchers, and to assist both parties in any part of the project related to PPI (including practical and administrative aspects). She is also responsible for communicating the interests, concerns, and feedback of the PRP group to the project management team.

PRPs have their own teleconferences (TCs), at least once a year, led by the PPI Coordinator with the minutes being shared with the consortium. PRPs are also invited to participate in TCs and face-to-face (F2F) meetings with researchers within the project. There is a large annual F2F project meeting, held in Europe, to which all consortium members are invited and PRPs are encouraged to attend. PRP travel, accommodation and subsistence are pre-booked by RTCure to facilitate attendance.

Initially PRPs were allocated to individual WPs based on their preferences, or if no preference was given then based on where they were needed. Each WP had at least one PRP allocated to it. However, this structure turned out to be unsatisfactory for the PRPs, as some felt isolated in their WP and thought they would benefit from discussing matters with their peers. Consequently, nine months into the project PRPs formed one PRP group which could be consulted by the consortium when required with researcher requests for PRP input/feedback being conveyed via the PPI Coordinator. Participation is voluntary at all times and the pooled PRP group allowed both cross-cover of PRPs when needed and more diverse input. Selected PRP contributions to RTCure until January 2021 are summarised at the end of Additional files 1 and 2. See Additional file 3 for the Guidance for Reporting Involvement of Patients and the Public-Long Form (GRIPP2-LF) [29] for RTCure.

At the outset of the project all PRPs were asked to enter a personal legal agreement with the consortium, put forward by a representative from the legal department of the consortium’s EFPIA coordinator and approved by RTCure project management. This consisted of 25 items in seven areas: ‘Subject matter of the agreement’, ‘Compensation’, ‘Confidentiality, archiving and data protection’, ‘Right to results’, ‘Compliance’, ‘Term’ and ‘Miscellaneous’, with the purpose of protecting the project and those involved in it.

However, this agreement had been adapted from the original consortium members’ agreement and therefore was lengthy, contained much legal terminology (which PRPs did not find easy to understand), put considerable personal liability on individual PRPs (who have no institutional indemnity) and therefore came to be regarded as inappropriate for use with PRPs. After prolonged investigations and discussions over a two-year period, this was replaced by a mutual non-disclosure agreement with the consortium (consisting of seven items solely around confidentiality), to be signed by PRPs prior to attending annual F2F meetings.

### Mid-term evaluation of PPI in RTCure

The PPI Coordinator, some PRPs and a researcher (WP7 lead) co-designed two surveys, one for researchers and another for PRPs, in order to evaluate PPI within the project. These used Likert-type scales and free text boxes, and were adapted (with permission) from response surveys previously used in EuroTEAM (an EU-funded project) [30]. Both surveys were circulated to a PPI task force (PRPs, researchers and industry) for agreement before being sent out for completion. The Researcher Survey consisted of 14 questions (see Additional file 1) and the PRP Survey of 19 questions (see Additional file 2). As an aide mémoire, a very brief summary of each WP and PRPs contributions to RTCure thus far was provided at the end of each survey.

Both online surveys were designed to allow anonymous responses and were distributed in January 2021 to 61 researchers and 9 PRPs by the PPI Coordinator using Google Forms, with a further email reminder sent out in February 2021. Due to a low response rate from researchers (9/61), the Researcher Survey was sent out again in April 2021 with a further reminder a week later. As this was a service evaluation, formal ethical approval was not needed for research of this kind, according to the UK Health Research Authority.

Quantitative data from both surveys were summarised using inbuilt Google Forms tools, and the free text exported into NVivo 12 Pro (QSR International, Burlington, MA, USA) software to assist qualitative analysis. A PRP with experience in qualitative methods (SdS) carried out an inductive thematic analysis of the data, whereby coding and theme development were driven by the content of the free text comments and not a pre-existing framework [31]. Based on the dataset, codes were systematically generated by SdS and a random sample of the coding (approximately 20%) checked by a second PRP also experienced in qualitative research (RW). Themes were identified by looking for recurring patterns in the data, and further refined by SdS and RW [32]. All authors agreed on the final themes.

## Results

### Researcher survey

Twenty participants completed the Researcher Survey (response rate 33%); 15 worked in academia and 5 worked for pharmaceutical companies. Nine researchers were clinical, 10 non-clinical and 1 participant was a medical statistician. Quantitative results from the Researcher (R) Survey are shown in Table 1.

**Table 1:** Quantitative results of the Researcher (R) Survey (Rs; *N* = 20)

Q1. Which type of organisation do you represent?								
	Academia	EFPIA	SME	Other				
Number (%) of Rs	15 (75%)	5 (25%)	0 (0%)	0 (0%)				
Q2. What is your position?								
	Clinical Researcher	Non-clinical Researcher	Other					
Number (%) of Rs	9 (45%)	10 (50%)	1 (5%)					
Q3. How much experience of working with patients/public as research partners did you have before your involvement with RTCure?*								
	No experience at all	Slight experience	Moderate experience	A good deal of experience	Extensive experience			
Number (%) of Rs	5 (25%)	4 (20%)	6 (30%)	2 (10%)	4 (20%)			
Q4. Which Work Package (WP) have you been most involved in?								
	WP1	WP2	WP3	WP4	WP5	WP6	WP7	
Number (%) of Rs	2 (10%)	2 (10%)	5 (25%)	4 (20%)	2 (10%)	3 (15%)	2 (10%)	
Q5. How much do you think that our patient/public research partners have been able to contribute to this Work Package?								
	No contribution at all	Minor contribution	Moderate contribution	Large contribution	Extremely large contribution			
Number (%) of Rs	3 (15%)	4 (20%)	5 (25%)	7 (35%)	1 (5%)			
Q6. Which other Work Package(s) have you been involved in (if any)?								
	WP1	WP2	WP3	WP4	WP5	WP6	WP7	None
Number (%) of Rs	0 (0%)	3 (15%)	2 (10%)	3 (15%)	2 (10%)	6 (30%)	0 (0%)	4 (20%)
Q7. How much do you think that our patient/public research partners have been able to contribute to this Work Package?								
	No contribution at all	Minor contribution	Moderate contribution	Large contribution	Extremely large contribution			
Number (%) of Rs	3 (15%)	5 (25%)	7 (35%)	5 (25%)	0 (0%)			
Q8. What kind of impact do you think patient and public involvement (PPI) has had on RTCure overall so far?^^^								
	Extremely small impact	Small impact	Moderate impact	Large impact	Extremely large impact			
Number (%) of Rs	0 (0%)	9 (45%)	2 (10%)	9 (45%)	0 (0%)			
Q9. Have you had any experience so far of working with Patient/Public Research Partners during RTCure?								
	Yes	No						
Number (%) of Rs	15 (75%)	5 (25%)						
Q11. Has your experience from working with PRPs in RTCure changed your views on working with PPI?								
	Yes	No	Don’t know					
Number (%) of Rs	6 (30%)	12 (60%)	2 (10%)					
Q12. Has your experience from working with PRPs in RTCure had any effect on your research perspective/thinking?								
	Yes	No						
Number (%) of Rs	12 (60%)	8 (40%)						

*EFPIA* European Federation of Pharmaceutical Industries and Associations, *SME* Small-to-medium enterprise

*One person chose two options. ^^^ ‘Moderate impact’ option erroneously written as ‘no impact’ on original survey; numbers given for ‘moderate impact’ have been adjusted based one person stating in the free text they wanted to choose ‘moderate impact’ and another person ticking both ‘small impact’ and ‘large impact’

Qs 10, 13 & 14 were purely qualitative questions

Six main themes, with some overlap between them, were identified from the qualitative data:

#### Theme 1: PRP contributions

It was widely recognised that PRPs had significantly contributed to RTCure. Contributions ranged from providing insights from their lived experience with RA to helping design the clinical trials and commenting on ethical aspects:

*The PRPs have provided important input as to the requirements for preventive interventions from the patient perspective, which guides the design of preventive intervention trials.* – R16 (Clinical Researcher, Academia)

*Provide feedback in regards to ethical aspects and direct patient insights into life with the disease.* – R4 (Non-clinical Researcher, Pharmaceutical Industry)

PRP input into how data are collected and shared was also seen as valuable:

*Extremely useful insights into patient perspectives on data collection and data sharing within the consortium.* – R2 (Clinical Researcher, Academia)

Highlighted as well was PRPs’ contribution to project outputs, with PRPs also sharing their own experiences from RTCure with the wider scientific community:

*Substantial contribution to discussions about balancing risk of interventions in ‘at-risk’ populations against benefits, according to individual-level probability of disease progression - which in turn fed into development of ‘Briefing Document’ [a document providing background information to the Swedish Medical Products Agency to inform a dialogue between RTCure partners and the Agency relating to prevention therapies for RA].* – R8 (Clinical Researcher, Academia)

*PRPs…have also presented a poster with the experiences from RTCure at EULAR [Congress 2020].* – R7 (Non-clinical Researcher, Academia)

It was also acknowledged that PRPs contributed more to clinical than non-clinical WPs. However, even PRPs sharing their experiences of living with RA had a broad effect across the project, particularly on non-clinical researchers:

*In general, I think the contribution of PPI is most considerable in clinical/clinical-orientated WPs. However, although perhaps not directly visible/tangible, contributions of patients during the RTCure meetings and elsewhere on how patients (have to) cope with the disease, is very important and relevant for basic researchers like myself. –* R19 (Non-clinical Researcher, Academia)

#### Theme 2: experiences of PPI

Almost all researchers shared positive experiences of PPI from the project, particularly when direct F2F interaction could take place:

*I think they are doing a great job…At the moments partners’ input was needed, they were always available.* – R6 (Clinical Researcher, Academia)

*At consortium meetings (i.e. pre-Covid), PRP involvement in discussion fora was really welcome and informed decision making, it seemed to me - about big picture issues.* – R8 (Clinical Researcher, Academia)

*This was mainly ‘over coffee’. I see these interactions as very positive*. – R19 (Non-clinical Researcher, Academia)

*There have been no negative experiences apart from their absence in our day-to-day activities - discussing socially during annual meetings and engaging in dedicated sessions has always been a positive experience.* – R13 (Non-clinical Researcher, Pharmaceutical Industry)

It was noted that when remote working became a necessity during the COVID-19 pandemic, the choice of teleconferencing platform affected the level of PRP participation:

*Zoom rather than [other] teleconferencing [platforms] appears to draw additional PRP input into meetings.* – R18 (Medical Statistician, Academia)

Only one researcher reported a wholly negative experience with a PRP, which was the result of a misunderstanding:

*I had sent out a questionnaire about available data sets that was sent to the entire RTCure email distribution list…which included the PRPs. Some of them filled out the questionnaire and ticked the box 'no data sets available' (as was to be expected!) but one PRP took offence at being asked to fill out the questionnaire. Of course, I had never expected any of the PRPs to contribute in this way and I had not realised they were on the email distribution list.* – R12 (Non-clinical Researcher, Academia)

Another researcher cited both positive and negative experiences:

*Positive: working and discussing with the PRP group, learning how to work together. Negative: the hard work with the legal agreement for participating in private-public partnerships, no support for the PRPs.* - R7 (Non-clinical Researcher, Academia)

Overall, PPI experiences reported by researchers were positive with the only real negative experience being the challenges of reaching consensus on the PRP legal agreement.

#### Theme 3: impact of PPI on RTCure

Some researchers were uncertain about the impact of PPI on the project. When asked how PRPs had contributed to their WPs, one researcher (R9) expressed being “Unsure” whilst another (R12) said “I don’t know”. Both were non-clinical researchers working in academia; R9 said they had not worked with PRPs in RTCure, whereas R12 indicated they had but had had a negative experience (see quote from Theme 2).

However, the majority of researchers felt that PPI had had a positive impact on RTCure, from influencing what was being looked at to directing the focus of the research, and could see real value in it:

*They are forcing us to constantly reflect on the impact of what we are doing; on at least one occasion they actually influenced the question we thought we needed to answer.* – R10 (Non-clinical Researcher, Pharmaceutical Industry)

*I could previously forget to take [into account] the patient views in some aspects, but now more regularly remember it in more situations (from planning of meetings to writing of reports).* – R7 (Non-clinical Researcher, Academia)

*Directing where the research focus should be. Hearing from patients is a critical component of understanding where unmet need is, and the therapeutics that will bring patient benefit.* – R14 (Non-clinical Researcher, Pharmaceutical Industry)

One researcher pointed out the importance of PPI in order to recruit people into clinical trials and translate any successful treatments into clinical practice:

*Insight from a public/patient perspective regarding the issues surrounding interventions in the pre-clinical state are absolutely essential to the future success of potential tolerising therapies. The issues involved are very complex, and without public/patient 'buy-in' it is extremely difficult to recruit to clinical trials in this area (and equally translate any future successful therapies to clinical practice).* – R20 (Clinical Researcher, Academia)

Also mentioned was how PPI had helped improve meetings, not just for PRPs but for everyone involved in the project:

*The PRPs have been contributing to creating better meeting conditions, both for physical meetings and meetings online.* – R7 (Non-clinical Researcher, Academia)

#### Theme 4: impact of PPI on researchers

As well as PPI impacting the project, it was clear that it had also had a personal impact on researchers themselves, particularly from hearing experiences of RA from people who live with the disease. This in turn affected how researchers would proceed going forward:

*I believe they [PRPs] play 2 important roles: first they bring an important perspective as to what matters to the people suffering from the disease. It is all too easy to forget that what we do has impact on real people and their presence is both a motivation and a constant reminder that whatever samples we are getting doesn't come from a model but from a real human being. Second…they force us to get out of our ivory tower and express our ideas in a clear and concise way.* – R5 (Non-clinical Researcher, Pharmaceutical Industry)

*Forces us as researchers to think how what we do impacts the patient…Strengthened my view of the importance of PPI.* – R18 (Medical Statistician, Academia)

*Hearing directly from a patient about their experience, and also what they observe about us as a research team/community. The latter drove home how important scientific communication to the wider public is a critical part of science and medicines research*. – R14 (Non-clinical Researcher, Pharmaceutical Industry)

Two researchers (both non-clinical working in the pharmaceutical industry) also mentioned how PPI made them revisit their assumptions on data sharing, particularly when working with industry:

*We often take consent for granted when it comes to data sharing, and it was important that PRPs expressed their concern regarding sharing data with commercial partners : this forced me to reconsider the reasons why they were concerned and to look beyond what was convenient and/or efficient toward what is respectful of everybody's feelings. –* R10 (Non-clinical Researcher, Pharmaceutical Industry)

*I was assuming most patients would consent to clinical analysis provided it would improve their condition, regardless of the partner involved. I was surprised at their lukewarm reaction to pharmaceutical companies getting access to their samples and data, and had to update my views after that.* – R5 (Non-clinical Researcher, Pharmaceutical Industry)

Through PPI within RTCure, there was also raised awareness amongst researchers about the value of PPI and barriers which PRPs can face:

*More aware of PRPs and what value they can add to a research project.* – R9 (Non-clinical Researcher, Academia)

*Open and frank discussion is key. PRPs are very good at pulling us up on things we miss, mis-interpret or do wrong…PRPs repeatedly open our eyes to things we haven't considered.* – R15 (Clinical Researcher, Academia)

*Realised some of the benefits of dedicated PRPs as well as the obstacles they face.* – R3 (Clinical Researcher, Academia)

#### Theme 5: influence on future projects

The way PPI had been handled in RTCure also made researchers think about how they would incorporate PPI into projects in future:

*Earlier engagement and somewhat larger groups of PRPs to capture different perspectives. –* R11 (Clinical Researcher, Academia)

*Underscored importance of integrating PPI from the earliest stages and throughout the project lifespan.* – R20 (Clinical Researcher, Academia)

*The importance of getting processes correct from the start (e.g. the [PRP] contract) has been highlighted.* – R2 (Clinical Researcher, Academia)

A common thread was the importance of involving PRPs as early as possible in projects and getting off to a good start. Also recognised was providing PRPs with a handbook, explaining the project and terms used, from the beginning so they are better able to participate:

*I would prepare a vade mecum of terms and concepts relevant to the project to be shared with non-specialists upfront and give some more room to PRPs during the kick-off meeting.* - R10 (Non-clinical Researcher, Pharmaceutical Industry)

#### Theme 6: how PPI can be improved

Several suggestions were made on how PPI could be improved during the remaining time of the RTCure project e.g., having PRP-specific deliverables, providing lay summaries, supporting PRPs more and providing them with feedback:

*With a dedicated PRP workshop in the beginning of the project and then annually to decide on the work with specific deliverables that the PRP group agree on. The best work would come out from ideas driven by the PRPs and supported by all.* – R7 (Non-clinical Researcher, Academia)

*I feel that the requirement to include a laymen's summary slide in the beginning of a presentation at RTCure meetings is very useful. It allows the PRPs to participate much better in the discussion. It was not reinforced, though, and many speakers did not include such a slide…the need to present and discuss data in such a way that patients can participate is important. It needs to be more strictly applied.* – R16 (Clinical Researcher, Academia)

*The consortium needs to support PRPs even more.* – R15 (Clinical Researcher, Academia)

*Feedback to PPI partners letting them know their contribution is valued, promoting further valuable contributions.* – R18 (Medical Statistician, Academia)

Also mentioned was the need to have more ‘at-risk’ PRPs involved in the project, as the clinical trial participants will be people identified as being at-risk of developing RA:

*Would be good to have some PRPs who are in an at-risk phase - I know this has been identified as a need and such PRPs are being sought.* – R2 (Clinical Researcher, Academia)

There was also reflection on what could have been done better, which again was about creating a more inclusive environment:

*We should have planned our science communication better and could have organized the same speed-dating session as we had between companies & research groups in the kick-off meeting to allow PRPs to get to know us.* – R10 (Non-clinical Researcher, Pharmaceutical Industry)

### Patient/Public Research Partner Survey

Seven PRPs completed the PRP Survey (response rate 78%). Quantitative results from the PRP Survey are shown in Table 2.

**Table 2:** Quantitative results of the Patient/Public Research Partner (PRP) Survey (PRPs; *N* = 7)

Q1. What kind of impact do you think patient and public involvement (PPI) has had on RTCure overall?*							
	No impact	Extremely small impact	Small impact	Moderate impact	Large impact	Extremely large impact	
Number (%) of PRPs	1 (14.3%)	1 (14.3%)	4 (57%)	0 (0%)	1 (14.3%)	0 (0%)	
Q2. Which Work Package have you been most involved in?							
	WP1	WP2	WP3	WP4	WP5	WP6	WP7
Number (%) of PRPs	0 (0%)	2 (28.6%)	0 (0%)	0 (0%)	1 (14.3%)	2 (28.6%)	2 (28.6%)
Q3. How much do you think you have been able to contribute to this Work Package?							
	No contribution at all	Minor contribution	Moderate contribution	Large contribution	Extremely large contribution		
Number (%) of PRPs	2 (28.6%)	3 (42.9%)	2 (28.6%)	0 (0%)	0 (0%)		
Q4. Which other Work Package(s) have you been involved in (if any)?^							
	WP1	WP2	WP3	WP4	WP5	WP6	WP7
Number (%) of PRPs	2 (28.6%)	3 (42.9%)	2 (28.6%)	2 (28.6%)	0 (0%)	4 (57.1%)	4 (57.1%)
Q5. How much do you think you have been able to contribute to this/these Work Package/s?							
	No contribution at all	Minor contribution	Moderate contribution	Large contribution	Extremely large contribution		
Number (%) of PRPs	2 (28.6%)	3 (42.9%)	2 (28.6%)	0 (0%)	0 (0%)		
Q6. Do you think that you have an overall idea/understanding of the goals of the RTCure project?							
	Do not understand at all	Understand a little	Moderate understanding	Understand a lot	Understand everything		
Number (%) of PRPs	0 (0%)	4 (57.1%)	3 (42.9%)	0 (0%)	0 (0%)		
Q7. Do you think that you understand how the goals of RTCure might be achieved?							
	Do not understand at all	Understand a little	Moderate understanding	Understand a lot	Understand everything		
Number (%) of PRPs	0 (0%)	5 (71.4%)	2 (28.6%)	0 (0%)	0 (0%)		
Q8. What do you think about the number of tasks for Patient/Public Research Partners (PRPs) in RTCure?^§^							
	Far too few	Too few	About the right number	Too many	Far too many		
Number (%) of PRPs	2 (28.6%)	1 (14.3%)	5 (71.4%)	1 (14.3%)	0 (0%)		
Q9. What do you think about information received on reports or general progress in RTCure?							
	Far too little information	Too little information	About the right amount of information	Too much information	Far too much information		
Number (%) of PRPs	2 (28.6%)	1 (14.3%)	4 (57.1%)	0 (0%)	0 (0%)		
Q10. How welcome do you feel your opinions were?							
	Not at all welcome	Not very welcome	Moderately welcome	Very welcome	Extremely welcome		
Number (%) of PRPs	0 (0%)	0 (0%)	5 (71.4%)	2 (28.6%)	0 (0%)		
Q11. How well do you feel PRP involvement was coordinated?							
	Not at all well coordinated	Not very well coordinated	Moderately well coordinated	Very well coordinated	Extremely well coordinated		
Number (%) of PRPs	1 (14.3%)	1 (14.3%)	1 (14.3%)	4 (57.1%)	0 (0%)		
Q12. What do you think about the amount/usefulness of feedback you received on the outcome of your contribution to different tasks?							
	Far too little feedback	Too little feedback	About the right amount of feedback	Too much feedback	Far too much feedback		
Number (%) of PRPs	1 (14.3%)	4 (57.1%)	2 (28.6%)	0 (0%)	0 (0%)		
Q13. How well do you think your attendance in RTCure meetings was facilitated?							
	Not at all well facilitated	Not very well facilitated	Moderately well facilitated	Very well facilitated	Extremely well facilitated		
Number (%) of PRPs	0 (0%)	0 (0%)	2 (28.6%)	5 (71.4%)	0 (0%)		
Q14. Has your interest in contributing to future research projects as a PRP changed due to your experience in RTCure?							
	Yes	No					
Number (%) of PRPs	3 (42.9%)	4 (57.1%)					
Q18. Has your involvement with the RTCure project had an impact on you, either positive or negative?							
	Yes	No					
Number (%) of PRPs	6 (85.7%)	1 (14.3%)					

*‘Moderate impact’ option erroneously written as ‘no impact’ on original survey; from the free text that followed it seems the person really did mean to choose ‘no impact’. ^^^Can choose more than one option. ^§^Two people chose more than one option

Qs 15, 16, 17 and 19 were purely qualitative questions

Six main themes, with some overlap between them, were identified from the qualitative data:

#### Theme 1: PRP contributions

Most PRPs felt they had made contributions to RTCure, with the following mentioned:

*What is visible…is the contribution on RTCure website, the participation at EULAR Congresses and the accepted poster, the questionnaire on the attitude of sharing patient data and biological samples, the questionnaire on different trial designs and cohorts and the questionnaire on animal models in basic research…PRPs were invited to contribute to the Glossary and the lay language presentations.* – PRP7

*Highlighting need for increased ‘at-risk’ Research Partner contribution. Raising the ethics of at-risk individuals being treated with therapies/novel therapies, and % risk acceptable…Importance of separation of at-risk individual from early RA patient and not to use the term ‘pre-RA’…Contribution to PRP meetings/tasks to develop whole group feedback on cohorts in studies…Discussion and actions around animal involvement in development of drug therapies and representation of patients’ perspective.* – PRP2

However, one PRP felt that PRPs were not being involved enough in the project and were therefore largely having to create work for themselves:

*Don't feel we are asked to do any useful things for the project. We seem to mainly be doing tasks relating to our own PRP agenda that we’ve come up with.* – PRP6

#### Theme 2: experiences of PPI

Both positive and negative experiences were reported by PRPs. Being accepted, by other PRPs and by researchers, and being listened to were identified as important positives:

*I feel fully accepted by all involved, especially my PRPs and Susanne [PPI Coordinator].* – PRP5

*All researchers were interested in hearing PRPs’ opinions whenever we had some input.* – PRP7

*…feedback from PRPs taken on board sincerely.* – PRP2

Positive experiences from F2F interactions with researchers were also highlighted, especially important as meetings went online once the COVID-19 pandemic reached Europe in Spring 2020:

*Camaraderie and informally engaging with social activities during annual meetings, corridor conversations with scientists and researchers.* – PRP2

*Felt welcomed at the annual meeting (many researchers came to engage in conversation at the face-to-face annual event).* – PRP7

*The face-to-face meetings, there I felt we could directly contribute…The teleconferences were difficult to follow let alone contribute to. Too technical [language].* – PRP3

Difficulty committing time to the project (due to illness and work commitments) was identified as a barrier to participation:

*I only had little time on my hands…to dedicate to my role of PRP in RTCure.* – PRP7

*Time scale is difficult as a patient as life often not so predictable…* – PRP2

*…unfortunately [meetings] mostly in my working hours.* – PRP5

Four PRPs highlighted the PRP Agreement they were asked to sign at the start of their involvement, and the way it was handled as a negative experience. Comments from two PRPs demonstrated the immediate and also ongoing impact of this:

*That horribly complex, unfair legal contract imposed on us…caused a lot of unnecessary stress and ill feeling.* – PRP6

*[Pharmaceutical company]’s handling of the PPI representatives at the start of RTCure [re: PRP Agreement] crushed so much trust and was so disrespectful and dismissive that I doubt it can be repaired. And no matter how well we try to improve on the way, we cannot make the start go away.* – PRP4

#### Theme 3: impact of PPI on RTCure

PRPs mentioned the following as some of the positive impacts PPI has had on RTCure: raising awareness of PPI, changing the language used and improving meeting conditions:

*
Some professionals being aware that patients 'add value'.* – PRP1

*The inclusion of some ACPA [anti-citrullinated protein antibodies] negative people at-risk, looking also at those with early RA, a change in the language e.g. 'people at-risk' rather than 'patients'.* – PRP6

*Impact around clinical trials for biologic therapies involving animals and need for patient representation into debates on this, not just clinicians vs anti-animal experimentation [activists]. Feedback and changes around suitable venues, structure of sessions breaks, PRP expenses and lay summaries.* – PRP2

However, there was uncertainty about the magnitude of impact of PPI on the project, and the extent to which this had genuinely influenced the project’s outputs:

*I answered ‘small’ [impact] because I’m not quite sure.* – PRP3

*I’m really not sure if PPI is influencing RTCure, but I hope so.* – PRP5

*Looking at the list of the PRPs' contribution so far, I would say they had a significant contribution, but I am not so sure about the impact…I am not sure of the concrete added value to researchers’ work.* – PRP7

#### Theme 4: impact of RTCure on PRPs

Being involved with RTCure had marked effects on PRPs. A positive example given was based on good feelings created from bonding with other PRPs, having good leadership and researchers being interested in what PRPs had to say:

*Meeting other PRPs, creating a bond with them, having a very good coordinator of the group and seeing some of the researchers’ enthusiasm in working with PRPs had a highly positive impact on me.* – PRP7

Other examples given highlighted the negative impacts on some PRPs, with them feeling unsupported and alone:

*I was close to a mental breakdown at the start of the project over the unreasonable PRP agreement we were asked to sign. A lot of pressure was being put to sign and anyone who resisted was made to feel like they were being 'difficult'. No support from the PRP Coordinator - the one person on the project who should have been looking out for PRPs'* interests*.* – PRP6

*It has dented me and taken away some of the spontaneous joy in working as a PRP. I have felt very alone and vulnerable as a lay person. I am more aware of power structures, undemocratic behaviour, and old bad habits in the world of research. I will be more careful as to what and who I accept to work with in future.* – PRP4

#### Theme 5: engagement with PRPs

There were mixed views on engagement with PRPs and coordination of PPI within the project. Two PRPs felt PPI was being led well:

*Susanne [PPI Coordinator] is doing a great job.* – PRP3.

*Susanne Karlfeldt [PPI Coordinator] plays an important role in supporting the PRPs and we convey a big THANK YOU to her!* – PRP7

Other PRPs felt communication was inconsistent and poor overall:

*Sometimes feels loses momentum and then engagement.* – PRP2

*Communication with PRPs failed from the start…Overall I think information and communication (lay or otherwise) is poor in this project.* – PRP4

Lack of feedback was also highlighted, which left PRPs unclear about the overall progress of the project and whether their contributions were making any difference:

*I have no idea about the general progress and where we are at the moment.* – PRP5

*Rarely hear anything. Largely unaware what is happening with the project.* – PRP6

*I would have been very happy to know the added value of PRPs' contribution to the research (to the activities in the WPs). I know we have contributed, but in what way our contribution has added to the large picture?* – PRP7

Two PRPs had views on the qualifications needed to lead PPI in this kind of project, suggesting the importance of someone with both a research background and considerable experience in PPI:

*It would be good if those in charge of PPI were closer to the research (one or two of the WP-leaders maybe?). It's important that the person leading the PPI…doesn't get caught between consortium and PRPs; this creates unnecessary waiting times, stems the workflow and saps energy and commitment.* – PRP4

*This is a big project and someone with a lot of experience working with PRPs on big projects should have been appointed to the role. Sometimes it feels more like the PRPs guiding the PRP Coordinator rather than the other way around.* – PRP6

Also mentioned was the lack of knowledge researchers had in how to involve PRPS:

*Less knowledge (on researchers' part) on how to involve and to facilitate patients' involvement in the WPs.* – PRP7

#### Theme 6: how PPI can be improved

Several suggestions were made on how PPI can be improved in the project such as more and better communication with feedback provided, better online meeting conditions, covering travel and health insurance costs for attending F2F meetings, and compensating PRPs for their time and expertise:

*RTCure partners engage more with PRPs.* – PRP1

*Comparing to other IMI initiatives…the number of tasks could be risen…What could be improved is the Minutes – some information is too scientific to understand…Creating one or two PowerPoint slides in lay language (at the beginning of a meeting)...Receiving more feedback on our contribution. – PRP7*

*Being involved more and listened to by different work packages, more breaks in online meetings, shorter PRP teleconferences, recruitment of more people ‘at-risk’… payment of travel/health insurance to facilitate in-person attendance, payment for our work. – PRP6*

In contrast, one PRP was happy with the situation commenting:

*For me personally, nothing must be improved.* – PRP5

There was also reflection by many that PRPs should have been involved from the conception of RTCure; which would have helped them make an informed choice regarding involvement, influence the research questions, and facilitated them having an equal status to researchers during the project:

*PRP involvement prior to starting project would have helped…I would ideally want to be involved from the point of conception of a project, so I could make an informed decision about relevance to me, agreement with concept and how comfortable I felt with key players.* – PRP2

*PRPs not being involved BEFORE the grant was given and starting on the project about 6 months in - too late to change many of the main ideas.* – PRP6

*Certain ground must be prepared during the very first planning phase and preferably with PRPs involved. If collaboration with PRPs as equals is what the project wants, then the industry and academia must prepare the ground for it beforehand, it will not come automatically.* – PRP4

## Discussion

This mid-term evaluation of PPI in RTCure provides useful insight into PPI within a large international project with academic and industry partners. Importantly it captures both researcher and patient perspectives and highlights areas which can be improved in this and future projects. Both surveys shared four themes (PRP contributions, Experiences of PPI, Impact of PPI on RTCure and How PPI can be improved), with theme 4 (Impact of PPI on researchers v Impact of PPI on PRPs) being very similar. The only difference in themes was researchers commenting on how PPI would influence their future projects and PRPs discussing their future PPI engagement (theme 5 from both survey results), though this difference can be largely accounted for by the line of questioning in each survey.

### PPI impact

It is important to highlight the distinction between PRP contributions and PPI impact, as the two can often be conflated. PRPs can make many contributions (e.g., by attending meetings or providing feedback on documentation) to a project, but these do not necessarily translate to being seen to have made a change (or impact) to the project or those involved. Our results show a discordance in perceived PPI impact on RTCure between responding researchers and PRPs. It is difficult to assess whether PRPs underestimate their impact or responding researchers overestimate the impact of PPI. The low response rate (33%) from researchers may have introduced a significant response bias. The rate is similar to other PPI survey-based research [30]. It would be helpful to know why most researchers did not respond and future research should address this issue.

Of those researchers who responded, a major positive impact was that over half (60%) said PRPs had affected their research thinking. PPI often produces impact by changing what researchers ‘think and do’ which in turn affects their research [33]. However, these changes are usually undocumented as researchers are trained to present objective accounts of their work (and therefore not delve into their subjective personal journeys) [33] or are unaware that their perspective has changed [34]. A recent documented example of PPI affecting the mindset of rheumatology researchers was reported by APPROACH (an IMI-funded clinical study) and was seen as an important patient contribution to the project [9].

Researchers in our survey mentioned how PPI had made them question their assumptions, especially around data sharing, and how PRPs see things which they may miss. PPI can fill gaps in the experiential knowledge of researchers and correct their assumptions [33]. Insights and learning from researcher experiences of PPI, just like with patient experiences of PPI, can help shape research processes used by others in the future [28].

It is a role of PRPs to make researchers aware of unhelpful practices, perceptions of their illness or use of language [9]. In RTCure, PRPs brought about a number of changes to researcher language, such as not using the word ‘patient’ to describe a person at-risk of developing RA (as they have not been diagnosed with an illness), not using the word ‘pre-RA’ to refer to the ‘at-risk’ state (as some of these people may never go on to develop RA) and not using the word ‘subject’ to refer to people enrolled in clinical trials (as this term is deemed offensive to many participants).

### Providing feedback and PPI training

Over half (57%) of PRPs indicated they received too little feedback on the outcomes of their contributions to the project, and in turn this influenced the extent to which PRPs felt that their contributions were meaningful, valuable, or impactful. Regular communication and feedback to PRPs is essential. This helps show respect, value, appreciation, builds confidence, increases learning and development, and can allow researchers to reflect on the impact of PPI [35]. As recognised by one of the researchers in our surveys, feedback to PRPs regarding the impact of their own contributions may also increase their motivation to remain involved and contribute further [35, 36].Table 2 shows that PRPs were mainly involved in clinical WPs. EuroTEAM also reported that PRPs found it harder to make meaningful contributions to laboratory-based WPs [30]. The greater contribution by PRPs to clinical versus non-clinical WPs was also recognised by a non-clinical academic researcher in their free text response, who felt it important to expose non-clinical researchers (especially early career researchers) to PPI so they gain a better appreciation of how the illness affects people whose samples they are working on.

Other reports have identified that involving the public in preclinical and laboratory-based research can be challenging [37, 38]. Non-clinical researchers are unlikely to have regular experience of meeting, talking with and working alongside people with the illnesses they study, and may therefore not feel comfortable in these interactions [38]. PPI training could be integrated into training for researchers, including non-clinical and laboratory-based scientists [39], and guidance has recently been made available on how to involve patients and the public in laboratory-based research [40].

### PRP legal agreement

Public contributors make an emotional as well as intellectual investment when engaging in PPI activities, and the ‘emotional fallout can be immense following a difficult experience’ [41]. The challenging experience with the PRP legal agreement at the start of the project hindered the building of constructive relationships during the crucial initial period when trust is normally established. Many PRPs were unwilling to enter into the initially proposed lengthy, complex legal agreement with the consortium. After extensive consultation by the PPI Coordinator with many other parties (including EULAR and the IMI), no similar legal contract was found and no such agreement was used in other EU/IMI projects such as EuroTEAM and APPROACH [9, 30].

Aside from the fact that PRPs were not able to understand the legal language of the proposed agreement, many felt it was inequitable and unnecessary; having worked on multiple previous research projects based on trust. Considerable PRP and PPI Coordinator time and energy were spent resolving this matter, which had a negative impact on some PRPs.

The meaningfulness and impact of PPI relies on trust existing between researchers and PRPs [41]. When researchers recognise the concerns of PPI contributors and demonstrate responsiveness, it lays a foundation of trust [42]. Looking to the future and increasing PPI in research, if complex legal agreements are deemed absolutely necessary, it may be that PRPs will need to be affiliated to and indemnified under the umbrella of larger research organisations involved in the study.

### PRP compensation and reimbursement

Another important area of learning during RTCure related to reimbursement of PRP expenses and consideration of payment for participation. An essential element of co-production is proper payment of public contributors [43]. It can be assumed by researchers or those in healthcare organisations that patients only want to volunteer or are just ‘happy to be involved’ [44]. Although money was allocated for travel, accommodation, meals and other out-of-pocket expenses, there was no money allocated in RTCure's budget to compensate PRPs for their time and expertise. When payment is made to all team members except PRPs, a power imbalance is created [44]. Without payment offered, it is likely that PRPs in a project will not be representative of the whole patient spectrum, as only those who can afford to volunteer will participate [44, 45].

Travel insurance costs to attend meetings abroad (in order to cover any urgent medical treatment required) should be reimbursed as standard on international projects and ideally paid upfront by project management. Costs associated with remote participation (e.g., telephone, broadband, computer hardware and software, electricity, printing) are often overlooked and need to be budgeted for and reimbursed to PRPs. Such ‘beyond the room’ processes have as much impact on PPI activities as activities that researchers consider to be ‘the’ involvement activity, and therefore require careful consideration [42]. Practical processes, such as how reimbursement occurs, give public contributors an idea of how authentic the commitment is from researchers to them and how much genuine value is placed on their experience and knowledge [42].

### Barriers to participation

Our mid-term evaluation shows that barriers to participation remain for some PRPs such as time commitment, meetings being held during working hours and too much use of scientific language without lay explanation. Concerns over time commitment, including finding it harder to commit to involvement as there may be worsening of health, and use of technical language have previously been raised by PRPs in other projects [39]. From the start of RTCure there have been periods of inactivity for some PRPs for health-related and other reasons. Having an initial larger group of PRPs enables input at all times, means PRPs can support each other and provides continuity for the WP leads.

Researchers need to support PPI through developing resources e.g., glossaries, lay summaries and background information on important project elements, as lay members cannot be expected to make informed decisions and meaningful contributions to research without an understanding of the relevant science [37, 38]. Researchers in RTCure are now encouraged to start presentations with a summary slide in lay language and an RTCure Glossary is being co-developed by researchers and PRPs. When researchers have to discuss their research in lay terms, it encourages more self-reflection on their work and leads to an inclusive environment, which encourages more discussion with other researchers in the room [9].

### Early PPI

As highlighted by our survey responses, PPI needs to be at the project onset rather than a later add-on. Ideally, there should have been PPI at the grant application stage when ideas were being formulated. It has previously been reported that involving patients too late i.e., after a project is funded, makes it more difficult for patients to change the project’s priorities and outcomes [46]. No rheumatology clinical trials, published between 2016 and 2020, reported patient involvement in the commissioning or undertaking stages [19]. Clearly this is an area which needs to be urgently addressed. We acknowledge that there is a challenge in paying for PPI at a pre-award stage, and suggest that universities and hospitals set money aside for this purpose.

The difficult situation with the PRP legal agreement may well have not occurred, had a PRP been engaged prior to the start of the project. If PRPs had been recruited earlier and taken part in the initial ‘speed dating event’ with researchers at the project kick-off meeting, it would have helped with relationship building and setting expectations. This was also recognised by researchers in our surveys and they said their experience with RTCure in this regard would shape how they approach PPI in future projects.

### Role of the PPI coordinator

The role of the PPI lead is to initially facilitate interactions between PRPs and researchers, to liaise with both parties and prompt researchers if they are not providing feedback [35]. In RTCure, PPI coordination was an add-on to the PPI Coordinator’s existing role as a university Research Coordinator and had no specific job description. A PPI lead who lacks experience or training, may reduce dialogue between PRPs and researchers, and hinder problem-solving. It is important that any consortium includes leaders with an interest in PPI to provide support to the PPI Coordinator when needed [33].

In a large-scale long duration project like RTCure, it is clear that a strong background in PPI is important to allow the PPI lead to successfully coordinate PRP activities, and to keep the PRP group together and engaged throughout. Maintaining contact with PRPs is important as without this there is a risk of detachment from the project [9]. PRPs also require support (both physical and emotional) as they live with debilitating conditions that take up a lot of their time and energy. Successful co-production requires time investment and emotional work in order to build relationships, however, this is often under-appreciated and under-resourced [43].

### Future plans

SK presented our evaluation results at the 4th RTCure Annual meeting held virtually in September 2021, followed by a discussion (facilitated by SK and KR) between the consortium and PRPs. As a result, we plan to (i) have sessions where researchers can present topics of interest to PRPs in an accessible manner; (ii) hold a session where researchers and PRPs discuss how to better communicate with each other (possibly assisted by an external facilitator); and (iii) set up a working group to create a ‘Terms of reference’ document that PRPs, EFPIA partners and researchers can agree as the basis for collaborative working with PRPs in future projects.

A final evaluation of PPI in RTCure will take place towards the end of the project. We plan to share all our findings with the IMI, EFPIA, EULAR and national patient organisations in order to improve future collaborations between researchers, industry and PRPs.

### Study limitations

Limitations of our evaluation included (i) a low researcher response rate (33%) despite email reminders; (ii) we did not receive feedback from the two PRPs who had limited involvement since the start of RTCure; (iii) the two erroneous options in both surveys for the question on how much impact PPI has had on RTCure (although we tried our best to adjust our interpretation of the quantitative data based on free text responses to this question, it is impossible to know how many more researchers and PRPs would have chosen the ‘moderate impact’ option had it been available); (iv) thematic analysis of the qualitative data was completed by two PRPs who co-created and completed the survey(s); and (v) we did not reach data saturation or triangulate our findings.

## Conclusions

PPI seemed to have a significant positive impact on RTCure and researchers themselves. However, when feedback is limited, PRPs can be less aware of their impact on research projects and feel remote from the process. Researchers can benefit from training in PPI, in particular around communication and parity of status, from the start of a project. It is better if PRPs are involved early when the study is being formulated. The role of the PPI Coordinator, in large multicentre projects like RTCure, is crucial to keeping PRPs informed and engaged throughout. PRPs should not have to enter complex legal agreements, and must be fully compensated and reimbursed for their participation.

A Plain English Summary of this paper is available—see Additional file 4.

## Supplementary Information

**Additional file 1.** Researcher Survey.

**Additional file 2.** Patient-Public Research Partner Survey.

**Additional file 3.** GRIPP2-LF.

**Additional file 4. **Plain English Summary.

## Data Availability

The datasets analysed during the current study are available from the corresponding author on reasonable request.

## Abbreviations

APPROACH: Applied public–private research enabling osteoarthritis clinical headway
COVID-19: Coronavirus disease 2019
EuroTEAM: Towards Early diagnosis and biomarker validation in Arthritis Management
EFPIA: European Federation of Pharmaceutical Industries and Associations
EU: European Union
EULAR: European League Against Rheumatism
F2F: Face-to-face
GRIPP2-LF: Guidance for Reporting Involvement of Patients and the Public Long Form
IMI: Innovative Medicines Initiative
PPI: Patient and public involvement
PREFER: Patient Preferences in Benefit and Risk Assessments during the Treatment Life Cycle
PRP: Patient/Public Research Partner
R: Researcher
RA: Rheumatoid arthritis
RTCure: Rheuma Tolerance for Cure
TC: Teleconference
WP: Work package
