# Supplementary material for: Patient and public involvement in an international rheumatology translational research project: an evaluation
Source: BMC Rheumatol. 2022 Oct 22;6:83. doi: 10.1186/s41927-022-00311-w (PMC9588249; doi:10.1186/s41927-022-00311-w)
Supplement: Supplementary file 2 — Additional file 2. Patient-Public Research Partner Survey. [file 41927_2022_311_MOESM2_ESM.pdf]

PRP questionnaire on PPI work in RTCure

We would like to capture your thoughts about Patient and Public Involvement (PPI) within the RTCure project over its 3.5 years duration. In particular, we are keen to understand what has gone well and what has not gone well so far and where we can improve during the remainder of the project.

Please use the possibility to comment and extend your answers using the comment option after each question. The more feed back we receive, the better we can understand what has gone well and what has not gone well and how we can improve.

At the end of the questionnaire you can find a list of the different WPs and a summary of PRP contribution so far.

*Obligatorisk

1. What kind of impact do you think patient and public involvement (PPI) has had on RTCure overall? *

- [ ] Extremely small impact
- [ ] Small impact
- [ ] No impact
- [ ] Large impact
- [ ] Extremely large impact

Please describe any impact you think PPI has had on RTCure overall

Ditt svar

2. Which Work Package have you been most involved in? (WP list at end of document) *

- ◯ WP1
- ◯ WP2
- ◯ WP3
- ◯ WP4
- ◯ WP5
- ◯ WP6
- ◯ WP7

3. How much do you think you have been able to contribute to this Work Package? *

- [ ] No contribution at all
- [ ] Minor contribution
- [ ] Moderate contribution
- [ ] Large contribution
- [ ] Extremely large contribution

Please describe how, if at all, you think you have been able to contribute to this Work Package

Ditt svar

4. Which other Work Package(s) have you been involved in (if any)? *

- [ ] WP1
- [ ] WP2
- [ ] WP3
- [ ] WP4
- [ ] WP5
- [ ] WP6
- [ ] WP7

5. How much do you think you have been able to contribute to this/these Work Package/s?) *

- ○ No contribution at all
- ○ Minor contribution
- ○ Moderate contribution
- ○ Large contribution
- ○ Extremely large contribution

Please describe how, if at all, you think you have been able to contribute to this/these Work Package/s

Ditt svar

6. Do you think that you have an overall idea/understanding of the goals of the RTCure project? *

- [ ] Do not understand at all
- [ ] Understand a little
- [ ] Moderate understanding
- [ ] Understand a lot
- [ ] Understand everything

Comments to your reply

Ditt svar

7. Do you think that you understand how the goals of RTCure might be achieved? *

- [ ] Do not understand at all
- [ ] Understand a little
- [ ] Moderate understanding
- [ ] Understand a lot
- [ ] Understand everything

Comments to your reply

Ditt svar

8. What do you think about the number of tasks for patient/public research partners (PRPs) in RTCure? *

- [ ] Far too few
- [ ] Too few
- [ ] About the right number
- [ ] Too many
- [ ] Far too many

Comment to your above reply

Ditt svar

9. What do you think about information received on reports or general progress in RTCure? *

- [ ] Far too little information
- [ ] Too little information
- [ ] About the right amount of information
- [ ] Too much information
- [ ] Far too much information

Please comment to your reply above

Ditt svar

10. How welcome do you feel your opinions were? *

- [ ] Not at all welcome
- [ ] Not very welcome
- [ ] Moderately welcome
- [ ] Very welcome
- [ ] Extremely welcome

Please comment on your above reply

Ditt svar

11. How well do you feel PRP involvement was coordinated? *

- [ ] Not at all well coordinated
- [ ] Not very well coordinated
- [ ] Moderately well coordinated
- [ ] Very well coordinated
- [ ] Extremely well coordinated

Please comment to your above reply

Ditt svar

12. What do you think about the amount/usefulness of feedback you received on the outcome of your contribution to different tasks? *

- [ ] Far too little feedback
- [ ] Too little feedback
- [ ] About the right amount of feedback
- [ ] Too much feedback
- [ ] Far too much feedback

Please comment on your above reply

Ditt svar

13. How well do you think your attendance in RTCure meetings was facilitated? *

- ○ Not at all well facilitated
- ○ Not well facilitated
- ○ Moderately well facilitated
- ○ Very well facilitated
- ○ Extremely well facilitated

Pleas comment on your above reply

Ditt svar

14. Has your interest in contributing to future research projects as a PRP changed due to your experience in RTCure? *

- [ ] Yes
- [ ] No

If yes, please describe in what way

Ditt svar

15. What has gone well in terms of PRP involvement in RTCure so far?

Ditt svar

16. What has not gone so well in terms of PRP involvement in RTCure so far?

Ditt svar

17. Can you suggest ways that PRP involvement in RTCure can be improved for the remainder of the project?

Ditt svar

18. Has your involvement with the RTCure project had an impact on you, either

[illegible]
positive or negative? *

- [ ] Yes
- [ ] No

If so, please describe the impact:

Ditt svar

19. Anything else (not already covered) you would like to mention?

Ditt svar

WP-list

WP-list

WP1. Management, coordination, dissemination and sustainability

The aim of WP1 is to ensure a proper functioning of the project in order to achieve the objectives, to complete the milestones in time, to secure the deliverables and to make sure that the consortium's contractual duties are carried out.

WP2. Cohorts and ethics

WP2 will provide a platform for the analysis of existing and future cohorts of individuals at-risk for developing RA. Next to clinical, also ethical and patient-centered considerations of the at-risk state will be addressed in this work package.

WP3. Mechanisms of Immune Tolerance

The aim of WP3 is to elucidate critical immune reactions driving chronic rheumatic inflammation and thereby define novel targets and pathways which could contribute to the therapeutic establishment of immune tolerance, and thus novel targets of therapy.

WP4. Technologies for monitoring the RA-associated immune state

This WP will provide a solid platform for immune monitoring. The tools, data and standard operating procedures (SOPs) developed within the WP will be applicable to diverse clinical trials, using different tolerising approaches performed by partners throughout the consortium.

WP5. Bioinformatics and data

Collaborating with the other WPs, WP5 will provide solutions to store, curate, access, analyse and visualize large and highly dimensional datasets generated as part of the RTCure consortium.

WP6. Clinical studies

In WP6 we aim to build on the data in the other WPs to be able to design efficient but informative experimental medicine studies of potentially tolerogenic therapies defined in collaboration with the other WPs.

WP7. Ethics requirements

This work package sets out the 'ethics requirements' that the project must comply with.

Ditt svar

Summary PRP contribution

PRP Contributions in RTCure until 2021-01

- Extensive discussions and meetings on the need or no need of a formal agreement for PRP participation in RTCure. PRPs gave thorough input to the final Agreement that was agreed upon by all parties in the end. However, this is still a provisional solution and further discussions and maybe instructions and directions from IMI are needed.
- Input to lay description of RTCure for the website
- Participation in three annual meetings, including WP-breakout meetings in WPs 2, 5, 6 and 7.
- Answering a questionnaire regarding the attitude on sharing patient data and biological samples. The feedback given and answers provided resulted in a report that has been a very important supporting document in the discussions following thereafter.
- Giving input to different kinds of trial designs and cohorts for clinical trials. As above, the input given has been very important and useful for the planning of clinical trials in the project.
- Replied to a questionnaire on animal models in basic research. Very helpful and enlightening for researchers working with animal models
- Participation in a workshop on immune tolerance
- Participation in the Technologies for monitoring the RA-associated immune state (WP3 and 4, online meeting)
- Participation in some WP6 online meetings
- Presentation on the patient perspective, international rheumatology meeting in Washington
- Participation in annual EULAR meeting 2018 and 2019, including WP2 meetings
- Poster accepted at the EULAR 2020 digital meeting
- Work on adapting reference cards from EULAR to facilitate PRP involvement in research projects
- Work on adapting and develop a glossary based on glossary from EuroTeam
- Input to RTCure newsletters

Ditt svar

Skicka

Skicka aldrig lösenord med Google Formulär

Det här innehållet har varken skapats eller godkänts av Google. Anmäl otillåten användning - Användarvillkor - Integritetspolicy

Google Formulär
