# Supplementary material for: Patient and public involvement in an international rheumatology translational research project: an evaluation
Source: BMC Rheumatol. 2022 Oct 22;6:83. doi: 10.1186/s41927-022-00311-w (PMC9588249; doi:10.1186/s41927-022-00311-w)
Supplement: Supplementary file 4 — Additional file 4. Plain English Summary. [file 41927_2022_311_MOESM4_ESM.docx]

**Plain English Summary**

This paper discusses an evaluation of Patient and Public involvement (PPI) so far in Rheuma Tolerance for Cure (RTCure); a large international research project which focuses on rheumatoid arthritis prevention. At the midpoint of the study, the PPI Coordinator, some Patient/Public Research Partners (PRPs) and a researcher developed two surveys that were sent to all researchers and PRPs in order to evaluate PPI up to that point in time. Data collected from the surveys were analysed, reflected upon and fed back to the consortium at its annual conference. There was much learning about practical steps that can be taken to enhance working with PRPs, alongside identification of barriers and pitfalls that should be avoided. Overall PRP input was felt to benefit the project, but the perception of the ‘value’ of PPI was rated lower by PRPs than by researchers. We discuss why this might be, and what changes might improve the experience of PPI and maximise the value of collaboration with PRPs in research.
